# Materialism Predicts College Students' Entrepreneurial Intention: A Serial Mediation Model

**DOI:** 10.3389/fpsyg.2022.864069

**Published:** 2022-06-02

**Authors:** Yanbin Li, Yue Zhang, Feng Zhang

**Affiliations:** School of Economics and Management, North China Electric Power University, Beijing, China

**Keywords:** materialism, entrepreneurial intention, achievement motivation, entrepreneurial attitude, theory of planned behavior

## Abstract

Entrepreneurship is perceived as a critical pillar for unemployment alleviation and economic growth, especially in the era of COVID-19, which highlights the importance of the entrepreneurial potential of college students. The current research focused on the role of personal values in the entrepreneurial process and investigated the relationship between materialism and entrepreneurial intention among college students. Few studies have been examined this relationship, and the underlying mechanisms were also not identified. From the perspective of personal value, we hypothesized that materialism could positively predict entrepreneurial intention. Moreover, based on McClelland's theory of need for achievement and the theory of planned behavior, a serial mediation model, with achievement motivation and entrepreneurial attitude as the mediators, was proposed. We conducted a correlational study on a sample of 1,002 Chinese university students to examine our hypotheses. They participated in an online survey and completed the measurement of entrepreneurial intention, entrepreneurial attitude, materialism, and achievement motivation. The hypothesized models were examined through serial mediation bootstrapping procedures. The results showed that materialism positively predicted college students' entrepreneurial intention, and this relationship was serially mediated through achievement motivation and entrepreneurial attitude. Materialism boosted college students' achievement motivation, which in turn was associated with a more positive entrepreneurial attitude and subsequently stronger entrepreneurial intention. The present research is the first to empirically examine this association's mechanism and establish a serial mediation involving achievement motivation and entrepreneurial attitude. For the theoretical contribution, the present research provides a more comprehensive picture of the role of personal values in entrepreneurship by complementing the effect of materialism. And regarding the practical implications, the present research implies the silver lining of materialism and points out a possible way to enhance college students' entrepreneurial intention, i.e., entrepreneurial education could take advantage of the characteristics of materialism and transform the “harmful” value into socially beneficial entrepreneurial intentions through enhancing their achievement motivation and positive attitude toward entrepreneurship.

## Introduction

A slew of studies and reports documented that the outbreak of COVID-19 led to skyrocketing unemployment worldwide (e.g., Kawohl and Nordt, 2020; Tamesberger and Bacher, 2020). Among them, young graduates who have just finished their studies and looked for their first job are one of the most vulnerable groups in the labor market (Lambovska et al., 2021). Research has shown that many American college students lost a job or offer due to COVID (Aucejo et al., 2020), and Chinese college students faced a similar difficult situation (Mok et al., 2021). Entrepreneurship is a vital force in the economy (Okpala, 2012), which can reduce unemployment and help economic growth (Carree and Thurik, 2010). In particular, entrepreneurship is often perceived as an effective solution to graduate unemployment (Awogbenle and Iwuamadi, 2010; Chigunta, 2017), highlighting the importance of improving college students' entrepreneurial intention (Bird, 1988; Kautonen et al., 2015).

Studies abound concerning the influencing factor of entrepreneurial intention. Recently, an increasing number of researchers advocated improving understanding of the role of personal values in entrepreneurial intention (Fayolle et al., 2014), and it has become a fast-growing area of entrepreneurship research (Hueso et al., 2021). Most studies were based on a theoretical perspective that explores how Schwartz's four dimensions of values influence entrepreneurial intentions. For example, studies have remarked that self-enhancement value (Liñán et al., 2016; Gorgievski et al., 2018; Karimi and Makreet, 2020) could positively predict entrepreneurial intention.

However, few studies have focused on the role of specific values that are prevalent in real life. Materialism, a personal value stressing the ownership of material wealth and economic success (Richins, 2004; Kasser, 2016), is prevalent worldwide (Siahtiri and Lee, 2019). More importantly, research has shown that the materialism of students increased over their college years (Jiang et al., 2021). Nevertheless, the effect of materialism on entrepreneurial intention and the psychological mechanism underlying the effect remains unclear. Figuring out the role of materialism in entrepreneurial intentions helps design programs to enhance the entrepreneurial intentions of college students based on their existing values. Therefore, the current research aims to address these issues by examining the relationship between materialism and entrepreneurial intention and further exploring the mechanism under this relationship.

## Theoretical Background and Hypothesis Development

### The Relationship Between Materialism and Entrepreneurial Intention

From the perspective of personal value, Schwartz's value theory (Schwartz, 1992) outlined a circular structure of personal values with two dimensions and four integrated values: on the one hand, self-enhancement vs. self-transcendence, and on the other hand, openness to change vs. conservation. Individuals with self-enhancement values strive to acquire prestige and social status, desire control over others and resources, as well as value showing competence and personal success according to societal standards. This conflicts with self-transcendence values that stress benevolence and universalism.

Schwartz's value theory further presumed that adjacently located values usually lead individuals to similar behaviors or decisions. Materialism is located near self-enhancement values but opposite to self-transcendent values (Burroughs and Rindfleisch, 2002; Kilbourne et al., 2005; Karabati and Cemalcilar, 2010; Dittmar and Isham, 2022). Previous studies have shown that entrepreneurial intention is correlated positively with self-enhancement values (Liñán et al., 2016; Gorgievski et al., 2018; Karimi and Makreet, 2020; Ammeer et al., 2021) but negatively with the work values that are similar to self-transcendent values (Lechner et al., 2018). In this sense, materialism may also be able to predict entrepreneurial intentions positively.

Moreover, according to Richins and Dawson (1992), “materialists place possessions and their acquisition at the center of their lives”, view these [possessions] as essential to their satisfaction and well-being in their life,” and “tend to judge their own and others' success by the number and quality of possessions accumulated” (Richins and Dawson, 1992, p.304). Obtaining more money is one of the primary goals of materialists, as it allows them to own more abundant and luxurious possessions (Kasser, 2016). They may prefer to work longer hours and earn more money rather than spending that time leisurely (Richins and Dawson, 1992) in order to raise their living standard (Sidhu and Foo, 2015). Meanwhile, entrepreneurship is typically perceived as a career path that realizes personal needs regarding income, status, and prestige (Hirschi and Fischer, 2013), implying a positive association between materialism and the college students' entrepreneurial intention. Actually, entrepreneurs from a wide range of countries tend to possess materialistic values (McGrath et al., 1992).

To our knowledge, only one study directly examined the relationship between materialism and college students' entrepreneurial intention, which found a positive but insignificant correlation (Fatoki, 2015). However, the sample size of this study was relatively small (*N* = 146), and only business school students were included, which may render the results inconclusive and require further investigation. Despite the lack of direct and conclusive evidence, several studies regarding materialism and entrepreneurship-related variables provide indirect evidence. It has been found that materialism was positively correlated with college students' interest in entrepreneurship (Frunzaru and Leovaridis, 2016) and female microentrepreneurs' entrepreneurial self-efficacy (Salim et al., 2020), which have a substantial effect on entrepreneurial intention (Zhao et al., 2005; Nowiński et al., 2019). Likewise, materialists were found to work hard to complete challenging tasks (Vohs et al., 2006), and starting a business can be viewed as a challenging task.

In short, based on Schwartz's value theory, the definition of materialism, and relevant studies mentioned above, we offer our first hypothesis,

*H1: Materialism could positively predict college students' entrepreneurial intention*.

### Achievement Motivation and Entrepreneurial Attitude as the Underlying Mechanisms

The mechanisms underlying the relationship between materialism and entrepreneurial intention also remained unclear. One possible mediator is achievement motivation. Achievement motivation is a subjective and internal drive to push individuals to pursue success and prompt them to reach the goals they perceive to be valuable (Stewart and Roth, 2007). Based on McClelland (1961) theory of need for achievement (nAch), achievement motivation is significantly related to entrepreneurial intention (e.g., Collins et al., 2004; Bhatti et al., 2021; Biswas and Verma, 2021), even singled out as the most prevalent predictor of entrepreneurship (Babb and Babb, 1992). Achievement motivation is not only positively linked to entrepreneurial behavior but also materialism (e.g., Zhang et al., 2020), especially when the goal one aims to achieve is economic goals (Sirgy et al., 2013). Materialism might activate high motivation and effort to achieve wealth (Larsen et al., 1999). The above analysis suggested that achievement motivation might be a mediator between materialism and entrepreneurial intention. That is, materialists may show a stronger need for achievement and thus be more inclined to star-up their own business.

Furthermore, according to the theory of planned behavior (TPB; Ajzen, 1991) that states intentions are affected and predicted by certain specific attitudes, the role of achievement motivation may need to be realized by enhancing entrepreneurial attitude. Entrepreneurial attitude refers to the degree to which the individual holds a positive personal valuation about being an entrepreneur (Liñán and Chen, 2009). Research-based on TPB has shown that entrepreneurial attitude could mediate the relationship between achievement motivation and entrepreneurial intention (Karimi et al., 2017; Maharani et al., 2020; Bagiş et al., 2022), which implies that entrepreneurial attitude might be another mediator that exists between achievement motivation and entrepreneurial intention.

Together with the evidence above, achievement motivation and entrepreneurial attitude seem to link materialism to entrepreneurial intention. Furthermore, borrowing from entrepreneurship research and TPB, literature has shown that entrepreneurial motivation and entrepreneurial attitude could serially mediate the effect of entrepreneurial education on entrepreneurial intention, i.e., entrepreneurial education heightened the entrepreneurial motivation, which next improved the entrepreneurial attitude and thus promoted college students' entrepreneurial intention (Mahendra et al., 2017). In other words, achievement motivation and entrepreneurial attitude do not occur simultaneously but sequentially. To explain such association from the personal value side, albeit without direct evidence, a study regarding online time players showed that materialism significantly led to the pursuit of achievement, and such motivation fully mediated the effect of materialism on attitude toward online games (Chang and Zhang, 2008). Therefore, we have reason to believe that achievement motivation and entrepreneurial attitude could sequentially explain the association between materialism and entrepreneurial intention.

In sum, based on the theories and studies discussed above, it appears that materialism may predict entrepreneurial intention through a sequential path of achievement motivation and entrepreneurial attitude: college students with a higher level of materialism may be more motivated to achieve success and further be more favorable toward entrepreneurship, thus more willing to become an entrepreneur. Hence, we hypothesized that,

*H2: Achievement motivation and entrepreneurial attitude serially mediate the relation between materialism and entrepreneurial intention of college students*.

### The Present Study

**Figure 1:**
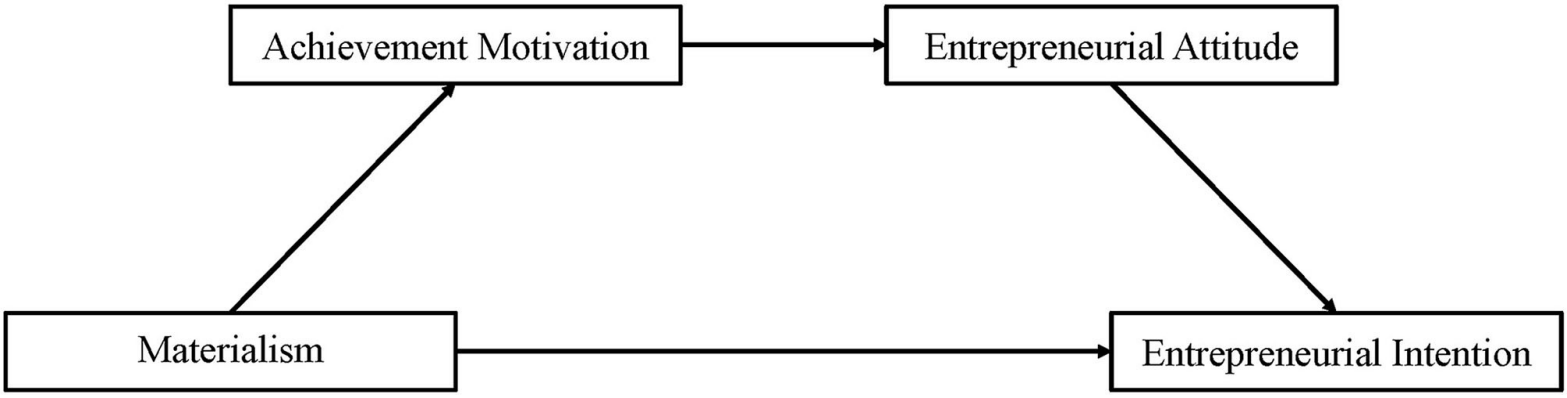
Hypothesized model of serial-multiple mediation of achievement motivation and entrepreneurial attitude in the relationship between materialism and entrepreneurial intention.

To sum up, the present study has two goals. First, we aimed to examine the relationship between materialism and entrepreneurial intention. Based on Schwartz's value theory, we hypothesized that materialism would positively predict entrepreneurial intention. Second, we aimed to explore the possible mechanism underlying the relationship between materialism and entrepreneurial intention. Based on McClelland's theory of nAch and TPB, we proposed a serial mediation model where achievement motivation and entrepreneurial attitude play as mediators sequentially in the association between materialism and entrepreneurial intention (see Figure 1). The conceptual model hypothesized that a higher level of materialism would be associated with stronger achievement motivation, which would then be associated with a more positive entrepreneurial attitude; the more positive entrepreneurial attitude would, in turn, be associated with a greater entrepreneurial intention. The hypothesized model was examined through an online survey with a large sample.

## Materials and Methods

### Participants and Procedure

Considering the impact of COVID-19, we collected data through an online survey questionnaire platform (https://www.wjx.cn). We designed and built the questionnaire on this platform. Only after all items had been completed could the participant submit the questionnaire. And owing to the IP restriction, one participant was only permitted to submit a reply once. The instruction for this survey was as below, “Thank you very much for taking the time to participate in this survey. Please fill out the following questionnaire according to your true feelings. The answer to a question is neither right nor wrong, nor good nor bad. Since the format of the questions in each section is not quite the same, please read the instructions in front of the questions carefully before you start answering them. According to the Statistics Act and the norms of scientific research, we will keep all the information collected confidential, including your personal information and your answers to the questions, and will not disclose them to outsiders.”

The sampling method employed for this study was cluster sampling and convenience sampling. Specifically, in order to enhance the sample diversity, the survey link is distributed through two channels. The first channel is sending the survey link to college counselors of various departments in one university. These counselors shared the link in class WeChat groups, and students were invited to complete the questionnaire online and voluntarily. The second channel is posting the survey link on the online survey platform where we designed our questionnaire. College student users of this platform voluntarily complete the questionnaire with a return of the website points as rewards. Data from 787 to 359 college students was collected separately through the two channels, i.e., a total of 1,146 Chinese college students voluntarily participated in this online survey. One hundred forty-four participants failed the attention check. Data from the remaining 1,002 participants (*M*_*age*_ = 21.58, *SD*_age_ = 3.16; 52.29% females) were analyzed. Details of sample demographics were as shown in Table 1.

**Table 1:** Demographic information of the current sample (*N* = 1,007).

**Demographic variables**	**Frequency**	**Percentage (%)**
Gender		
Male	478	47.71
Female	524	52.29
Grade		
Freshman	188	18.76
Sophomore	183	18.26
Junior	179	17.86
Senior	187	18.66
Postgraduate	265	26.45
Discipline of Students' Majors		
Science and engineering	232	23.15
Economics and management	548	54.69
Literature and history	179	17.86
Arts	24	2.40
Others	19	1.90
Family income level^a^		
Lowest	63	6.29
Lower-middle	228	22.75
Middle	527	52.59
Upper-middle	175	17.47
Highest	9	0.90

*^a^Family income level measured by a single question, i.e., “What is your family's current income level in the local area?”*.

### Measures

As in previous studies (e.g., Zhao et al., 2021), a short version of the scale was chosen to measure the research variables in order to reduce participants' dropout rates and increase their involvement with the survey. All items for measures were administrated in Chinese. The scales developed initially in English have been translated into Chinese, and the reliability and applicability of the translated version have been verified in previous studies (Tang and Tang, 2007; Hu et al., 2014; Wang et al., 2017). The scales are arranged in the following order: the measure of entrepreneurial intention, the measure of entrepreneurial attitude, the measure of achievement motivation, and the measure of materialism. Moreover, similar to the previous studies (e.g., Bergenholtz et al., 2021; Tufa, 2021), we adopted the original number of scale points for each measure, i.e., entrepreneurial intention and entrepreneurial attitude were measured on a 7-point Likert scale, and materialism and achievement motivation were measured on a 5-point Likert scale. Using different numbers of scale points in one survey is helpful to reduce common method bias (Jordan and Troth, 2020).

#### Entrepreneurial Intention

Entrepreneurial intention was measured using the 5-item scale developed by Chen et al. (1998). All the items were rated on a 7-point Likert scale (1 = totally disagree, 7 = totally agree). Sample items include “I am going to try hard to set up my own business and “I have been preparing to set up my own business.” The scores of five items were accumulated and averaged. A higher score indicated a stronger entrepreneurial intention. Cronbach's alpha was 0.94 in our sample.

#### Materialism

A 3-item version of the Material Values Scale (Richins, 2004) was used, a 5-point Likert scale ranging from 1 = totally disagree to 5 = totally agree. The three items are “I admire people who own expensive homes, cars, and clothes,” “I like a lot of luxury in my life,” and “I'd be happier if I could afford to buy more things.” Higher average scores for the three items indicated higher levels of materialism held by the participants. Cronbach's alpha for the scale was 0.70.

#### Achievement Motivation

We adapted four items from Tang and Tang (2007) measure of achievement motivation (e.g., “I desire to achieve a higher position for myself in society,” and “I hope to fulfill a personal vision”). Similarly, the participants responded to each item using a 5-point Likert scale (1 = totally disagree, 5 = totally agree). The total scores were also averaged, and higher scores indicate higher levels of achievement motivation. Cronbach's alpha for the scale was 0.78 in this sample.

#### Entrepreneurial Attitude

The entrepreneurial attitude was measured on a 7-point Likert scale (1 = totally disagree, 7 = totally agree) with the 5-item questionnaire developed by Liñán and Chen (2009) (e.g., “Being an entrepreneur implies more advantages than disadvantages to me” and “A career as an entrepreneur is attractive for me”). The total scores were also averaged, and a higher average score indicated a more positive entrepreneurial attitude. Cronbach's alpha for the scale was 0.93 in this sample.

#### Control Variables

Following the practice of previous studies, the participants' gender (Nowiński et al., 2019) and family income (van der Zwan et al., 2016) are likely to exert potential influences on our dependent variable—entrepreneurial intention. Thus, they were treated as covariates when examining the hypotheses. A single item measured family income (What is your family's current income level in the local area?) answered on a 5-point scale (1 = the lowest level, 5 = the highest level).

### Statistical Strategies

#### Discriminant Validity

We conducted confirmatory factor analyses (CFAs) to test the discriminant validity of the measured variables and the common method variance, using Mplus version 8.0. Regarding the discriminant validity, we first built a four-factor model (i.e., entrepreneurial intention, entrepreneurial attitude, materialism, and achievement motivation) and explored its psychometric properties, including comparative fit index (CFI), Tucker-Lewis index (TLI), standardized root mean square residual (SRMR) and root mean square error of approximation (RMSEA) as previous studies (e.g., Ding and Yu, 2021; Su, 2021). Then, we built three competitive models by combining the correlated factors into one and compared them with our hypothesized model. We did not rely upon chi-square as it has been found to be too sensitive to sample sizes over 250 (Bentler and Bonett, 1980). Following the suggestions of previous studies, CFI values > 0.95, TLI values > 0.90, SRME value <0.08 (Hu and Bentler, 1999; Hu et al., 2014) and RMSEA values <0.08 (MacCallum et al., 1996)indicated an acceptable model fit.

#### Common Method Variance

Since the current study deployed self-report questionnaires, it is essential to examine whether serious common method bias existed (Hariguna, 2021). Following the suggestion of Podsakoff et al. (2003), an unmeasured latent method factor method was applied to test the degree of common method bias. We constructed a latent method factor and loaded the method factor on all indices of entrepreneurial intention, materialism, achievement motivation, and entrepreneurial attitude. Suppose the five-factor measurement model regarding the common method factor and the four key research variables did not exhibit a significantly better fit to the data than the four-factor measurement model including four key variables. In that case, it turns out that the common method bias of the current study does not pose a severe threat to our results.

#### Hypothesis Examination

In terms of hypothesis examination, we first conducted linear regression analyses to test the main effect of materialism on entrepreneurial intention **(i.e., Hypothesis 1)**. We included control variables to diminish the spurious effects.

Next, Hayes (2017) statistical techniques (PROCESS v3.3) were employed to examine serial mediation **(i.e., Hypothesis 2)**. We fitted the theoretically-indicated serial mediation models (i.e., achievement motivation as the first mediator and entrepreneurial attitude as the second mediator) using PROCESS Model 6. Bootstrapping (5,000 resamples) was used to generate bias-corrected 95% confidence intervals (CIs) for the magnitude of all mediating effects. An effect was considered significant if the 95% CI did not include zero. Additionally, the above serial mediation model was tested with and without the control variables (i.e., college students' gender and family income level).

## Results

### Discriminant Validity and Common Method Variance Test

Before testing our hypotheses, we conducted a series of CFAs to evaluate the goodness of fit for the measurement model and the discriminant validity of the concepts. Following the cutoffs for the acceptable fit mentioned above (CFI > 0.95, TLI > 0.90, SRMR and RMSEA <0. 08), the results of CFA revealed acceptable psychometric properties of our 4-factor measurement model (see Table 2). The chi-square difference values with one degree of freedom (ranging from 375.01 to 1259.21) were all significant, indicating that the 4-factor model yielded the best fit.

**Table 2:** Results of confirmatory factor analyses (CFA).

**Model**	**χ^2^**	***df***	**CFI**	**TLI**	**RMSEA**	**SRMR**
4-factor	685.14	109	0.95	0.94	0.07	0.06
*inclusive materialism, achievement motivation, entrepreneurial attitude*,						
*entrepreneurial intention*						
3-factor	1558.31	112	0.89	0.86	0.11	0.09
*inclusive materialism, entrepreneurial intention, achievement motivation +*						
*entrepreneurial attitude*						
2-factor	1831.74	114	0.86	0.84	0.12	0.10
*inclusive materialism, entrepreneurial intention + achievement motivation +*						
*entrepreneurial attitude*						
1-factor	2322.50	115	0.83	0.79	0.14	0.11
*inclusive materialism + entrepreneurial intention + achievement motivation +*						
*entrepreneurial attitude*						

*CFI, comparative fit index; TLI, Tucker-Lewis index; RMSEA, root mean square error of Approximation; SRMR, standardized root mean square residual*.

Next, we used the unmeasured latent method factor method to test the degree of common method bias. The results demonstrated that the five-factor model regarding the common method factor and key variables (χ^2^ = 901.11, *df* = 97, CFI = 0.94, TLI = 0.91, RMSEA = 0.09, SRMR = 0.20) did not exhibit a better fit to the data than the four-factor measurement model including four key research variables. Therefore, the CMV of this study does not pose a serious threat to our results.

### Hypothesis Testing

#### Preliminary Analyses

We conducted Pearson's correlation analyses to see how the study variables were associated. Table 3 shows a significantly positive association between college students' materialism and entrepreneurial intention, providing initial evidence for Hypothesis 1. Moreover, in line with expectations, both achievement motivation and entrepreneurial attitude were positively correlated with materialism as well as entrepreneurial intention. At the same time, achievement motivation was also positively associated with an entrepreneurial attitude. These findings met the prerequisites for conducting hypothesis testing.

**Table 3:** Descriptive statistics and zero-order correlations of variables.

	***M***	***SD***	**1**	**2**	**3**	**4**	**5**	**6**
1. Gender^a^	0.48	0.50	–					
2. Family income	2.84	0.82	0.09**	–				
3. Materialism	3.26	0.84	−0.03	0.02	–			
4. Entrepreneurial intention	3.72	1.68	0.33***	0.31***	0.08*	–		
5. Achievement motivation	4.00	0.62	0.13***	0.13***	0.32***	0.36***	–	
6. Entrepreneurial attitude	4.46	1.50	0.30***	0.26***	0.15***	0.80***	0.45***	–

*** p <0.001, ** p <0.01, * p <0.05. N = 1,002. Gender ^a^ (0 = female, 1 = male).

#### Testing the Main Effect

As shown in Table 4 (Model 1), after controlling for gender and family income level, materialism still positively predicted entrepreneurial intention (β = 0.08, *t* = 2.94, *p* = 0.003, 95% CI = 0.06, 0.28), supporting Hypothesis 1. Next, we conducted a serial mediation model with gender and family income level as the control variables, materialism as the independent variable (IV), entrepreneurial intention as the dependent variable (DV), achievement motivation as the first mediator (M1), and entrepreneurial attitude as the second mediator (M2).

**Table 4:** Results of the proposed serial mediation model.

	**Model 1**		**Model 2**		**Model 3**		**Model 4**	
	**(Outcome: EI)**		**(Outcome: AM)**		**(Outcome: EA)**		**(Outcome: EI)**	
	**β**	***SE***	**β**	***SE***	**β**	***SE***	**β**	***SE***
**Control variables**								
Gender ^a^	0.31***	0.03	0.13***	0.03	0.23***	0.03	0.10***	0.02
Family Income	0.28***	0.03	0.11***	0.03	0.19***	0.03	0.10***	0.02
**Main effects**								
Materialism	0.08**	0.03	0.32***	0.03	0.02	0.03	−0.03	0.02
AM	–	–	–	–	0.39***	0.03	0.00	0.02
EA	–	–	–	–	–	–	0.75***	0.02
								
*F*	80.58***		52.34***		105.63***		394.12***	
*R* ^2^	0.20		0.14		0.30		0.66	

*** p <0.001, ** p <0.01. N = 1,002. Gender ^a^ (0 = female, 1 = male).

*AM, achievement motivation; EA, entrepreneurial attitude; EI, entrepreneurial intention*.

#### Testing the Serial Mediation Model

Results of the two-path serial mediation model are presented with standardized regression coefficients in Table 4 (Model 2–Model 4). The direct path from materialism (IV) to achievement motivation (M1) was significant (β = 0.32, *t* = 11.01, *p* < 0.001, bootstrapped 95% CI = 0.27, 0.38), indicating that the college students with higher materialism had stronger motivation to gain achievement. Meanwhile, the path from achievement motivation (M1) to entrepreneurial attitude (M2) was also significant (β = 0.39, *t* = 13.63, *p* < 0.001, bootstrapped 95% CI = 0.33, 0.45), demonstrating that students with stronger achievement motivation has more positive attitude toward entrepreneurship. The path from entrepreneurial attitude (M2) to entrepreneurial intention (DV) was significant (β = 0.75, *t* = 34.25, *p* < 0.001, bootstrapped 95% CI = 0.71, 0.79), revealing that students who has a more positive attitude toward entrepreneurship are more willing to start up their own business. However, the path from achievement motivation (M1) to entrepreneurial intention (DV) was not significant (β = 0.00, *t* = 0.01, *p* = 0.99, bootstrapped 95% CI = −0.04, 0.04). Similarly, the direct paths from materialism (IV) to entrepreneurial attitude (M2) (β = 0.02, *t* = 0.79, p = 0.43, bootstrapped 95% CI = −0.03, 0.07) and intention (DV) (β = −0.03, *t* = −1.44, *p* = 0.15, bootstrapped 95% CI = −0.07, 0.01) were not significant.

**Figure 2:**
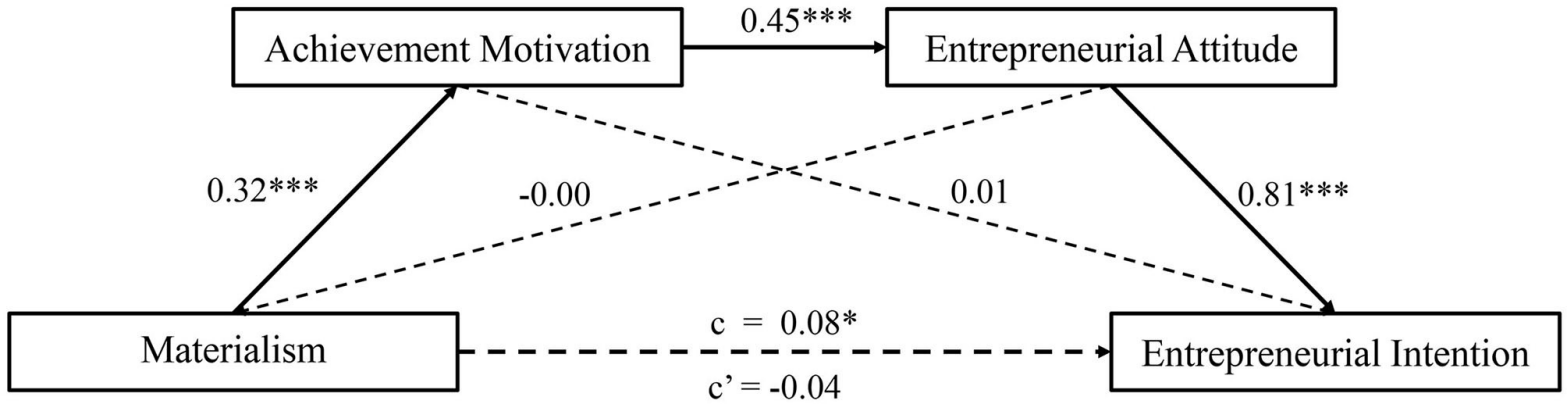
The serial mediation model without control variables. Path coefficients are standardized estimates. [c] represents the total effect, [c′] represents the direct effect. *p < 0.05; ***p < 0.001.

Figure 2 illustrates the serial mediation model without control variables. Overall, the results without control variables did not change meaningfully compared to those with control variables.

All indirect pathways calculations, including bias-corrected bootstrap 95% CIs, with and without control variables, are presented in Table 5. After controlling for gender and family income level, the total indirect effect was 0.11 (bootstrap 95% CI = 0.06, 0.16), and the proposed sequential mediation effect was 0.09 (bootstrap 95% CI = 0.07, 0.12, supporting Hypothesis 2. Alternative indirect pathways such as materialism through achievement motivation and materialism through entrepreneurial attitude were not significant, indicating that neither achievement motivation nor entrepreneurial attitude could solely explain the relationship between materialism and entrepreneurial intention. Similarly, the results without control variables did not change meaningfully.

**Table 5:** Indirect effects of materialism on entrepreneurial intention.

**Pathway**	**Effect**	**Boot SE**	**Boot LLCI**	**Boot ULCI**
*With control variables*				
Total	0.11	0.03	0.06	0.16
Materialism → AM → EI	0.00	0.01	−0.01	0.01
Materialism → EA → EI	0.02	0.02	−0.03	0.06
Materialism → AM → EA → EI	0.09	0.01	0.07	0.12
*Without control variables*				
Total	0.12	0.03	0.06	0.18
Materialism → AM → EI	0.00	0.01	−0.01	0.02
Materialism → EA → EI	0.00	0.02	−0.05	0.05
Materialism → AM → EA → EI	0.12	0.02	0.09	0.15

*AM, achievement motivation; EA, entrepreneurial attitude; EI, entrepreneurial intention*.

Given a significant indirect effect but no significant direct effect, the above results indicate that the relationship between materialism and entrepreneurial intention is fully mediated by achievement motivation and entrepreneurial attitude in a sequential manner. The serial mediation model with control variables explained overall the 66% of the variance (65% without control variables) of entrepreneurial intention.

#### Alternative Models

In an alternative model in which the order of the sequential mediators was switched, the serial mediation effect (materialism → entrepreneurial attitude → achievement motivation → entrepreneurial attitude) was no longer significant, with values of 0.00 (bootstrap 95% CI = −0.003, 0.003), even controlling for gender and family income level (effect = 0.00, bootstrap 95% CI = −0.003, 0.003). Thus, the order of the proposed mediators is meaningful, such that achievement motivation precedes entrepreneurial attitude in the meditational pathway.

## Discussion

Across the world, COVID-19 has resulted in an increase in graduate unemployment (Shahriar et al., 2021). It is often perceived that entrepreneurship among college students is an effective remedy for graduate unemployment (Awogbenle and Iwuamadi, 2010; Chigunta, 2017). Considerable agreement exists about the critical role of entrepreneurial intentions in the decision to start a new firm (e.g., Liñán et al., 2011; Fayolle and Liñán, 2014; Ozaralli and Rivenburgh, 2016; Meoli et al., 2020). Therefore, numerous studies explored the possible factors that could increase or decrease entrepreneurial intention. The current research focused on materialism, a personal value prevalent worldwide but often viewed as a “bad apple,” and revealed that materialism could positively predict college students' entrepreneurial intention. Such association achieves through strengthening achievement motivation and further promoting a positive entrepreneurial attitude, i.e., the college students' materialistic value could translate into high achievement motivation, which in turn is positively related to a favorable entrepreneurial attitude and subsequently higher entrepreneurial intention. The current research implies the silver lining of materialism and points to a new approach to increase entrepreneurial intention, which previous studies have overlooked.

### Materialism and Entrepreneurial Intention

From the perspective of personal value, materialism is located in close proximity to self-enhancement values when mapping it onto Schwartz's value circle model (Burroughs and Rindfleisch, 2002; Kilbourne et al., 2005; Karabati and Cemalcilar, 2010). Self-enhancement values have been viewed as a positive factor in entrepreneurial intention (Liñán et al., 2016; Gorgievski et al., 2018; Karimi and Makreet, 2020). Schwartz's value circle model presumed that adjacently located values usually lead individuals to similar behaviors or decisions (Schwartz et al., 2012). Previous studies supported this presumption by indicating that work values found near self-enhancement values, such as stressing salary and prestige (Hirschi and Fischer, 2013) or the importance of extrinsic awards (e.g., promotion, Lechner et al., 2018), were related to higher entrepreneurial intention. In line with these studies, the present research also provided supportive evidence.

The present research documented that the materialistic value could positively predict entrepreneurial intention as its “neighbors.” Materialists attach importance to acquiring money and material possessions; material possessions are central to their life and happiness; the more possessions they own, the more successful they perceive themselves (Richins and Dawson, 1992). These characteristics lead materialists to an inclination toward a high-paying, high-status job when choosing a career (Kasser, 2002). That is to say, the students with higher materialism have a stronger desire for money, which makes them prefer a career choice that could bring them a high income and thus be better able to indulge in a taste for acquisition. Staring own business is typically described as such a kind of career choice (Hirschi and Fischer, 2013). In this way, materialism would yield an increase in willingness to launch their own venture and consider entrepreneurship as a career option.

Moreover, in line with the previous study (Fatoki, 2015), the present research also identified a positive relationship between materialism and entrepreneurial intention. However, the association found in the current research is significant, which is different from the previous study. The difference in sample size may cause this discrepancy. The sample size of the Fayolle et al. (2014) study may not be adequate, which may lead to the significant results disappearing. Actually, the correlation found in the current study was relatively small (*r* = 0.08). One possible explanation is that materialism might be a distal factor in entrepreneurial intention rather than a proximal one. The previous study has demonstrated that the total effect of distal factors is minor or even subtle compared to proximal factors, and the effect of distal factors needs to be transmitted through mediators (Shrout and Bolger, 2002). Our results regarding the serial mediation model supported the above explanation: the effect of materialism needs to be transmitted through achievement motivation and entrepreneurial attitude. Only when college students' materialism could activate achievement motivation and achievement motivation subsequently lead to a positive attitude toward entrepreneurship could materialism better increase college students' entrepreneurial intention. Otherwise, the effect of materialism might be relatively small, albeit still significant.

### The Serial Mediation Model

Another finding of the current research is that achievement motivation and entrepreneurial attitude could explain the association between materialism and entrepreneurial intention. That is, a serial mediation model has been established. It is noteworthy that although the results met the requirements for “full mediation” (i.e., a significant indirect effect but no significant direct effect of materialism on entrepreneurial intention), we do not claim a “full mediation” because the claim of full mediation would likely discourage future research from examining other possible mechanisms and constrain theory development (Rucker et al., 2011).

Consistent with previous studies (Larsen et al., 1999; Chang and Zhang, 2008; Sirgy et al., 2013), the current research indicated that materialism is related to higher achievement motivation. The students who hold a higher level of materialism may put material possessions at the center of their lives and set material acquisition as their personal vision (Richins and Dawson, 1992). To acquire luxurious possession as much as possible, they may need to reap wealth, achieve a higher position in society, and make an effort to achieve their vision. In short, an emphasis on material possessions will motivate college students to seek wealth, place high importance on income and lead them to prioritize the satisfaction gained from the achievement of personal interests, thus exhibiting higher achievement motivation. In line with McClelland (1961) theory of nAch, having a need for achievement motivates entrepreneurship. Achievement motivation brings a positive attitude toward entrepreneurship, as reported in earlier studies (e.g., Kusmintarti et al., 2014; Ajiwibawani and Subroto, 2017; Maharani et al., 2020; Bagiş et al., 2022). College students with higher achievement motivation may feel choosing entrepreneurship as a career is more advantageous and attractive and expect becoming an entrepreneur would entail great satisfaction. Finally, such positive inclination formed a willingness to star-up their own business, which coheres with the prediction of TPB (e.g., Liñán et al., 2011; Anwar et al., 2021). In brief, materialism increases entrepreneurial intention through achievement motivation and entrepreneurial attitude.

Besides the serial mediation, the current research also indicated that achievement motivation and entrepreneurial attitude could not independently mediate the relationship between materialism and entrepreneurial intention after controlling the other mediator. This finding suggests that achievement motivation and entrepreneurial attitude might be interdependent when transmitting the effect of materialism to entrepreneurial intention. First, concerning achievement motivation, it is more correlated with entrepreneurial attitude than with intention. And the entrepreneurial attitude was strongly associated with entrepreneurial intention. Both reasons may render the direct effect of achievement motivation on entrepreneurial intention nonsignificant (Rucker et al., 2011). Thus, achievement motivation could not play an independent mediating role when controlling entrepreneurial attitude. A similar case can be seen in Thelen (2019). Second, a possible reason why entrepreneurial attitude cannot independently mediate the effect of materialism when controlling achievement motivation is that materialism is still a distal factor in entrepreneurial attitude. A piece of evidence is the small correlation between materialism and entrepreneurial attitude (*r* = 0.15). As discussed in the prior section, the effect of distal factors needs to be transmitted *via* mediators (Shrout and Bolger, 2002). Studies regarding self-enhancement values also documented that the direct effect of self-enhancement values on entrepreneurial attitude was insignificant (Gorgievski et al., 2018; Sánchez-Báez et al., 2018), which implied that the effect of materialism on entrepreneurship-related variables might need to be realized through mediators, such as achievement motivation. In other words, achievement motivation can be viewed as a bridge between materialism and entrepreneurial activity.

### Theoretical Implications

The current research identified a positive association between materialism and college students' entrepreneurial intention with a large sample, which contributed to the existing literature concerning the role of personal values in entrepreneurial activity by complementing the effect of materialism. Previous studies on personal values mainly focused on Schwartz's four integrated values or their related work values (Hueso et al., 2021). Although materialism partly overlaps with self-enhancement values, it has its own unique features. For example, materialists value power, achievement, salary, and prestige because they view them as tools to acquire more and better material possessions instead of the ultimate goals. Materialists attach importance to the number of material possessions and the symbolic role of acquiring and possessing material wealth for the self and others (Shrum et al., 2013). Therefore, investigating the relationship between materialism and entrepreneurial intentions can provide a more comprehensive picture of the role of personal values in entrepreneurship.

Furthermore, the current research is the first to empirically examine the psychological mechanism underlying the relationship between materialism and entrepreneurial intention and establish a serial mediation involving achievement motivation and entrepreneurial attitude. In addition, most previous studies merely relied on TPB to identify the mediators in the relationship between personal values and entrepreneurial intention (e.g., Gorgievski et al., 2018; Kruse et al., 2019; Yasir et al., 2021), neglecting the role of motivation in the relationship of values-entrepreneurship. By integrating the theory of nAch and the TPB, the present research pointed out a potential path from personal value to the entrepreneurial intention where motivation and attitude both play a significant role, which underlines the impact of motivation and helps break down the barriers between different theories.

### Practical Implications

The current research also has practical implications for decreasing materialism and increasing entrepreneurial intention. First, regarding materialism decrease, materialism is often considered a detriment to “good stuff” such as self-esteem and well-being (for a review, see Kasser, 2016). Hence, a slew of studies has sought ways to reduce materialism through education or intervention programs (e.g., Kasser et al., 2014; Chaplin et al., 2019; Unanue et al., 2021). The present study, however, illustrated an alternative to interventions that could also render materialism harmless. That is, owing to the feature of materialism that places high importance on acquiring money and material possessions, materialism could translate into the high entrepreneurial intention of college students through enhancing their achievement motivation and positive attitude toward entrepreneurship. It is critical to note that we are not encouraging enhancing materialism; instead, we suggest the possibility that educators could take advantage of the characteristics of materialism and transform the “harmful” value into a socially beneficial factor, for example, entrepreneurial intentions.

Second, concerning entrepreneurial intention promotion, earlier studies focused on cultivating students' abilities or traits that they do not possess or need to develop, such as creativity (e.g., Hu et al., 2018; Tantawy et al., 2021) or proactive personality (e.g., Crant, 1996; Neneh, 2019). The current research pointed out another way—leveraging students' existing values to develop entrepreneurial intention. During the university years, students will be engaged in the process of identity construction (Arnett, 2000) that will aid them in clarifying their values. A longitudinal study concerning Chinses college students indicated that materialism showed an increasing trajectory over the college years (Jiang et al., 2021). The growing trend of college students' materialism implied materialism might be an ever-increasing “resource” of entrepreneurial intention. In addition, the current research describes how to use this resource, i.e., translating students' materialistic value into achievement motivation and enhancing their positive entrepreneurial attitude.

In other words, the current research suggests that entrepreneurial education could utilize the current materialistic value of students to increase their entrepreneurial intention. For example, with the help of specific software (Hsueh, 2018; Hananto, 2021) or algorithms (Astuti and Handoko, 2018; Imron and Kusumah, 2018), educators could identify and target the students who hold high materialism through, as well as predicting the likelihood of them starting a business in the future through machine learning (Jen and Lin, 2021; Prayitno et al., 2021; Saputro and Nanang, 2021; Sugiyanto, 2021). And then, entrepreneurial education programs could provide them with decision support systems (Azis et al., 2020; Fujishima, 2022), e-learning classes (Widiyanto et al., 2021), and customized training plans (Thelen, 2021) to improve their achievement motivation and enhance attitudes and skills regarding entrepreneurship.

### Limitations and Future Research Recommendations

Notwithstanding its contributions, the present research has some limitations. First, the current research only adopted a cross-sectional design, which is hard to draw causal conclusions. Future research could employ a longitudinal design and measure the research variables at different time points (e.g., Hamid et al., 2022) or conduct a laboratory experiment and prime or manipulate students' materialistic value (e.g., Wang et al., 2019), to verify the causal relationship between materialism and entrepreneurial intention.

Second, the present research merely measured the general entrepreneurial intention. In other words, we did not distinguish between the tendency to start a commercial enterprise and that to create a non-profit organization (referred to as social entrepreneurship, Peredo and McLean, 2006). The positive effect of materialism may only exist in the former. That is, materialists may be more willing to become business entrepreneurs rather than social entrepreneurs. Future research could explore the association between materialism and social entrepreneurial intention.

Finally, the current research did not explore possible moderators. Both environmental (e.g., entrepreneurial munificence; Tang and Tang, 2007) and individual factors (e.g., risk preference; Barbosa et al., 2007) may moderate the effect of materialism on achievement motivation or entrepreneurial intention. Future research could establish a moderated mediation model or a moderated serial mediation model to exhibit the mechanisms underlying the association between materialism and entrepreneurial intention with greater comprehensiveness and depth.

## Conclusion

Overall, the current research identifies materialism as a new positive predictor of entrepreneurial intention and is the first to establish a serial mediation model between materialism and entrepreneurial intention. The current research demonstrates that college students' materialism could increase their entrepreneurial intention by strengthening their achievement motivation and entrepreneurial attitude, i.e., materialism boosted college students' achievement motivation, which in turn was associated with a more positive entrepreneurial attitude and subsequently stronger entrepreneurial intention. These findings pointed out that, albeit often regarded as a “bad apple,” materialism could still exert a positive effect on entrepreneurial intention through certain means, which implies the silver lining of materialism and delineates a possible way to enhance entrepreneurial intention based on the existing value of college students.

## Data Availability Statement

The raw data supporting the conclusions of this article will be made available on request without undue reservation. Further inquiries can be directed to the corresponding author.

## Ethics Statement

The studies involving human participants were reviewed and approved by the Ethics Committee of the School of Economics and Management of North China Electric Power University. Written informed consent for participation was not required for this study in accordance with the national legislation and the institutional requirements.

## Author Contributions

YL, YZ, and FZ conceived the research and developed the theoretical framework. YL collected the data, secured funding, and administered the project. YZ performed statistical analyses and wrote the original draft. All authors contributed to the manuscript revision and approved the submitted version.

## Funding

This study was supported and funded by the National General Project of the National Education Science 13th Five-Year Plan (National Social Science Foundation of China, No. BIA190186).

## Conflict of Interest

The authors declare that the research was conducted in the absence of any commercial or financial relationships that could be construed as a potential conflict of interest.

## Publisher's Note

All claims expressed in this article are solely those of the authors and do not necessarily represent those of their affiliated organizations, or those of the publisher, the editors and the reviewers. Any product that may be evaluated in this article, or claim that may be made by its manufacturer, is not guaranteed or endorsed by the publisher.
